# Editorial comment: Urorectal fistula repair using different approaches: operative results and quality of life issues

**DOI:** 10.1590/S1677-5538.IBJU.2020.0476.1

**Published:** 2021-02-03

**Authors:** André G. Cavalcanti

**Affiliations:** 1 Universidade Federal do Estado do Rio de Janeiro Departamento de Cirurgia Geral e Especializada Rio de Janeiro RJ Brasil Departamento de Cirurgia Geral e Especializada, Universidade Federal do Estado do Rio de Janeiro - UNIRIO, Rio de Janeiro, RJ, Brasil

## COMMENT

The urethral-rectal fistula (URF) is a devastating condition for the patient, especially when associated with the treatment of prostate cancer, where we often observe a healthy patient and with the expectation of a curative treatment. As discussed in this study, the treatment of urethral-rectal fistulas involves a series of decisions: use conservative treatments, necessity of a colostomy, timing for a definitive surgical treatment and choice of the best surgical approach.

The authors observed the results of the URF surgical treatment in a retrospective review of 39 patients operated at 3 institutions, and was possible a late evaluation for the uniform measurement of the results, through specific questionnaires (1). Patients were treated using a variety of techniques: abdominal (10.5%), abdominal-perineal (15.8%), perineal (29%), posterior trans-sphincteric (34.2%), anterior trans-sphincteric sagittal (5.3%), trans-coccygeal (2.6%) and trans-anal endoscopic microsurgery (2.6%). Another important observation is the fact that 9 (23%) of the patients received treatment after surgical failure, with no description as to whether this specific group was treated using one of the predominantly surgical techniques. This diversity can somehow hinder the comparative analysis of the results.

The authors presented a high success rate for surgical treatment, 89.5% and show no association between failures and the surgical technique, which confirms the observation of high success rates can be achieved regardless of the surgical technique if it is performed by a team with experience in the management of this type of pathology. As discussed in the study, multivariate analysis of factors related to failure due to the limited sample of patients is practically impossible.

The analysis of erectile dysfunction (ED) and urinary incontinence (UI) and their relationship with the treatment of URF is difficult in a retrospective study without adequate application of symptom questionnaires before the procedures. In addition, the causal effect of cancer treatment and URF treatment is difficult to measure. Especially for urinary incontinence, its evaluation in a patient with URF is extremely difficult. Despite this, it is clear in the analysis of the results that a significant number of patients will require further treatment to manage UI and ED, and this should be considered in the technical planning of the initial treatment.

The authors analyzed the cases of perineal and abdominal-perineal approaches in the same group and compare them with trans-sphincter approaches with more satisfactory results on the preservation of erectile function and satisfaction (statistically limited) for this second group. From my point of view, the perineal approach is completely different in terms of aggressiveness and potential damage when compared to abdomino-penrineal approaches and therefore should not be compared together as proposed by the authors. Final conclusions about the ED result scan only be obtained in prospective comparative studies, but we know that are difficult to be realized for a relatively unusual type of pathology.

Despite the observations, it is a very useful study to provide a broad view of the possibilities of treatment of URF, showing that in experienced hands, suitable results can be expected regardless of the surgical technique. The present study has some limitations as part of the data was obtained retrospectively. However, to my knowledge this is the first study to address the findings and outcomes of PF specifically and exclusively with non-sexual etiology in a latin american country. Congratulation to the authors.
